# Influence of subject characteristics on DTI parameters in the normal heart

**DOI:** 10.1186/1532-429X-16-S1-P7

**Published:** 2014-01-16

**Authors:** Laura-Ann McGill, Pedro Ferreira, Andrew D Scott, Sonia Nielles-Vallespin, Tevfik Ismail, Ranil Silva, Philip J Kilner, David Firmin, Dudley J Pennell

**Affiliations:** 1 The Cardiovascular BRU, Royal Brompton Hospital, London, UK; 2 National Heart, Lung and Blood Institute., National Institute of Health, Bethesda, Maryland, USA

## Background

Myocardial disarray is considered an important histological feature of hypertrophic cardiomyopathy (HCM)[1]. *In vivo *Cardiac Diffusion Tensor Imaging (cDTI) offers the potential to detect myocardial disarray and describe mean intravoxel myocyte orientation; however to date there is little quantitative cDTI data in the normal heart. In this study, we aim to establish the impact of physical and cardiac characteristics on cDTI parameters in a cohort of healthy volunteers.

## Methods

We recruited 46 healthy volunteers for cDTI at 3T. Three short axis mid-ventricular slices were acquired with multiple breath holds at the systolic pause. Data was post-processed with a platform, developed in-house, to create Fractional anisotropy (FA), Mean diffusivity (MD) and Helical Angle (HA) maps.

## Results

**Figure 1:**
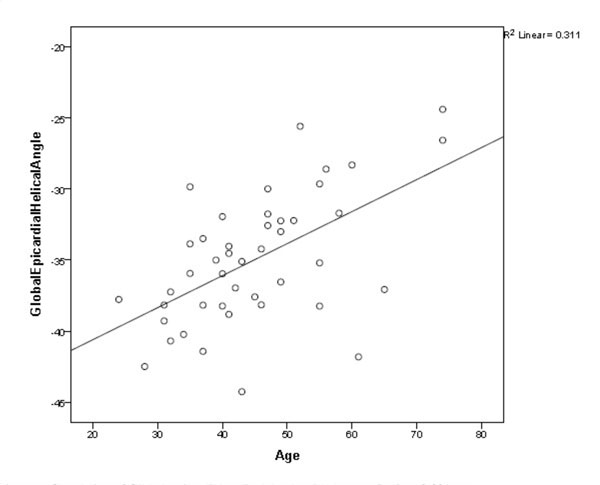
**Correlation of Global Epicardial Helical Angle with Age (r = 0.56; p < 0.001)**.

Two of the original 46 volunteers were excluded due to ECG irregularities. Data was successfully acquired in the remaining 44 volunteers (table 1). FA, MD and global endocardial HA values were found to be independent of subject age, sex, body surface area (BSA), indexed left ventricular end diastolic volume (LVEDVi) and indexed left ventricular mass (LVMi). There was a significant correlation between age and the global epicardial HA (r = 0.56, p < 0.001) with a gradual decline in leftward HA angulation with increasing age (Figure 1). This correlation persisted after adjusting for ventricular length (r = 0.45, p = 0.002) and BSA (r = 0.42, p = 0.004). There were also significant correlations between global epicardial HA and lateral mitral annular plane systolic excursion (MAPSE) (r = -0.473, p 0.001), and between lateral MAPSE and age (r = -0.375, p = 0.012) suggesting that the association between age and HA may be mediated through age related reduction in lateral MAPSE.

**Table 1:** 

Baseline Characteristics	N = 44
Age: yrs (range)	45 (24-74)

Male subjects	27 (61%)

BSA: m^2^	1.88 ± 0.20

BMI: kg/m^2^	24.5 ± 2.95

LVEDVi: mL/m^2^	77.5 ± 13.9

LVMi: g/m^2^	64.0 ± 17.0

LVEF: %	68 ± 6

Ventricular length in diastole: mm	95.2 ± 8.56

	

**DTI parameters**	

MD global: ×10^-3^mm^2^/s	0.93 ± 0.14

FA global	0.47 ± 0.05

Endocardial HA global:°	34 ± 5

Mesocardial HA global:°	-2 ± 3

Epicardial HA global:°	-35 ± 5

Mean ± SD

## Conclusions

Our data suggests that the cDTI parameters FA and MD, which describe intravoxel myocyte organisation and average myocardial diffusivity respectively, are independent of physical characteristics in healthy subjects. However, there is an association between age and epicardial HA which may be the result of age related loss in longitudinal function[2]. Future work will consider the importance of technical factors such as a SNR and myocardial strain, which may vary with body habitus.

## Funding

NIHR cardiovascular BRU Royal Brompton Hospital & Imperial College.

## References

[B1] Maron BJ Circulation 1979 59 689 706 10.1161/01.CIR.59.4.689 570464

[B2] Innelli Eur J Echocardiogr 2008 9 241 249 17586096 10.1016/j.euje.2007.03.044

